# SMILE: systems metabolomics using interpretable learning and evolution

**DOI:** 10.1186/s12859-021-04209-1

**Published:** 2021-05-28

**Authors:** Chengyuan Sha, Miroslava Cuperlovic-Culf, Ting Hu

**Affiliations:** 1 School of Computing, Queen’s University, Kingston, ON Canada; 2 Digital Technologies Research Center, National Research Council Canada, Ottawa, ON Canada

**Keywords:** Metabolomics, Alzheimer’s disease, Interpretable machine learning, Feature interaction, Evolutionary algorithm

## Abstract

**Background:**

Direct link between metabolism and cell and organism phenotype in health and disease makes metabolomics, a high throughput study of small molecular metabolites, an essential methodology for understanding and diagnosing disease development and progression. Machine learning methods have seen increasing adoptions in metabolomics thanks to their powerful prediction abilities. However, the “black-box” nature of many machine learning models remains a major challenge for wide acceptance and utility as it makes the interpretation of decision process difficult. This challenge is particularly predominant in biomedical research where understanding of the underlying decision making mechanism is essential for insuring safety and gaining new knowledge.

**Results:**

In this article, we proposed a novel computational framework, Systems Metabolomics using Interpretable Learning and Evolution (SMILE), for supervised metabolomics data analysis. Our methodology uses an evolutionary algorithm to learn interpretable predictive models and to identify the most influential metabolites and their interactions in association with disease. Moreover, we have developed a web application with a graphical user interface that can be used for easy analysis, interpretation and visualization of the results. Performance of the method and utilization of the web interface is shown using metabolomics data for Alzheimer’s disease.

**Conclusions:**

SMILE was able to identify several influential metabolites on AD and to provide interpretable predictive models that can be further used for a better understanding of the metabolic background of AD. SMILE addresses the emerging issue of interpretability and explainability in machine learning, and contributes to more transparent and powerful applications of machine learning in bioinformatics.

**Supplementary Information:**

The online version contains supplementary material available at 10.1186/s12859-021-04209-1.

## Background

In recent years, the availability of large datasets and the exponential advancing in computing power led to a rapid growth of machine learning applications in a variety of fields. Partial linear regression (PLR), artificial neural network (ANN), support vector machine (SVM), evolutionary algorithm and random forest (RF) have been widely used for metabolomics analysis [1]. Simple linear methods are more interpretable but do not perform as well. More complex learning methods, such as ANN and ensemble learning, can provide high prediction accuracy but are almost impossible to interpret [2]. These models remain mostly “black boxes” where the insights about the data and the working mechanisms of decision making are hidden in increasingly complex structures of the models. For deep neural networks, one needs numerous parameters to describe the model and it is impossible to entirely understand its mechanistic under-working [3, 4].

Understanding why a decision has been made is critical to gain users’ trust, which is fundamental in fields like bioinformatics. One important means to understand how and why a machine prediction has been made is to investigate what variables or features contribute to that decision-making, either through individual effect or interaction with one another. Novel approaches for interpretable machine learning with better information about the feature interactions would be particularly beneficial for analysis of omics data.

In particular, more advanced analysis methods for high throughput metabolic data with its closest link to actual cellular phenotype are highly desirable. Metabolomics combined with appropriate analytical methodologies can provide both biological knowledge, leading to novel therapeutic approaches, as well as biomarker panels, aimed towards early diagnosis of significant phenotypic changes. Neurodegenerative disorders, including dementia and Parkinson’s disease, are characterized by the progressive degeneration of the structure and function of the central or peripheral nervous system. Role of metabolic changes in the development and progression of these diseases is increasingly recognised (recently reviewed in [5, 6]).

Currently, world-wide around 50 million people are living with dementia and about 10 million new cases are being diagnosed every year. Alzheimer’s disease (AD) is the most prevalent age related dementia characterized, at the late stages, by the dysfunction and loss of synapses and eventual neuronal death induced by an accumulation of senile plaques and neurofibrillary tangles in the brain [7]. The symptoms of AD include memory loss, difficulty completing familiar tasks and personality changes. AD is a progressive neurodegenerative disease however causes of AD are still not fully understood. Genetically, $\epsilon 4$ allele of apolipoprotein E gene is widely accepted as a major genetic risk factor for AD [8] with APOE$\epsilon 4$ leading to an increased risk and APOE$\epsilon 2$ suggesting a decreased risk relative to the most common version of APOE$\epsilon 3$ [9]. APOE$\epsilon 4$ has been linked to the reduced efficiency in several brain pathways including as examples lipid transport and glucose metabolism. Recently, fructose metabolism in the brain has been proposed as a possible mechanism driving AD [10]. Dysfunction of many other metabolic pathways have been outlined as part of AD development and progression including changes in the metabolism of glucose [11], insulin [12], ketones [13], oxidative stress [14, 15], fructose [10], and bile acids [16]. Vitamine D [17] has, for example, been indicated as highly relevant in AD even suggesting that AD is a modern disease driven by changes in dietary lifestyle and its essence a metabolic disease [18]. Although all these and many other metabolic changes have been observed in AD patients or models it is still not clear what are the major disease drivers and early changes leading to this disease. Early diagnostic markers that can indicate AD related changes prior to symptom development and can show patients who will progress from mild cognitive impairment (MCI) stage to AD are sorely needed.

Metabolomics is the scientific study of chemical processes involving low-molecular-weight molecules, which include lipids, amino acids, peptides, sugars, bile acids and organic acids. These metabolites are the result as well as drivers of processes that are actually occurring in the biological systems and are the footprint of complex biological processes as well as a reflection of the well-being of our body. By quantitatively studying metabolites and comparing body-fluid samples from phenotypically distinguished populations, researchers are able to better understand the pathology of complex diseases [19, 20]. A number of recent studies have found that amino acids, glycoproteins, and lipids were significantly altered in AD patients [21, 22]. Advanced machine learning techniques can help identify novel metabolic markers and links between metabolites in the disease development and progression leading to more informative, early markers for AD. Several recent reviews described different application of machine learning in metabolomics in some detail [23–25]

In this article, we propose a new interpretable machine learning framework for metabolic data analysis. It uses an evolutionary algorithm to learn compact and interpretable predictive models and uses an ensemble of evolved models to identify the most potentially influential metabolites and their interactions associated with AD. Our bioinformatics results provide new insights into the disease and generate hypotheses for further biological investigations. All source code to implement our method is publicly available. Moreover, in order to facilitate an easy adoption of our methodology and to benefit a larger research community, we developed a web interface that interprets and visualizes the learning results. We studied a published AD metabolomic dataset. Our approach was able to identify both known and novel metabolites and metabolite interactions linked to the disease. Our results are expected to provide not only new insights into AD but also a powerful computational tool for metabolomics research.

## Methods

### Metabolomic data on AD

In this research, we analyzed a metabolomic dataset on AD from a study published and described in detail by Wang et al. [26]. The dataset includes 57 patients with AD, 58 patients with amnestic mild cognitive impairment (aMCI, which is considered as an early form of AD), and 57 healthy individuals as controls. Fasting venous blood was collected from all the participants. The plasma samples were then analyzed using ultra-performance liquid chromatography-time-of-flight mass spectrometry and gas chromatography-time-of-flight mass spectrometry providing concentrations of 242 plasma metabolites (including fatty acids, amino acids, nucleic acids and carbohydrates).

Prior to applying the machine learning analysis, we normalized the concentration levels of metabolites to be within the range of $[-\,1, 1]$, using the *MinMaxScaler* method from *Scikit-learn* Python library [27].

### Systems metabolomics using interpretable learning and evolution (SMILE)

#### Overview of SMILE

We propose a computational framework for metabolomics data analysis, Systems Metabolomics using Interpretable Learning and Evolution (SMILE). SMILE uses an evolutionary algorithm for learning interpretable predictive models, provides explanations of its decision-making, and identifies key metabolites and their interactions associated with a complex trait. In order to benefit a wider research community, we also developed a web application for SMILE with a graphical user interface, where researchers can perform interpretable machine learning analysis and visualize the results of their own metabolomic data. The source code of SMILE and the metabolomic data used in this study are publicly available at https://github.com/MIB-Lab/SMILE, the detailed documentation of function usage in SMILE is avaliable at https://smile-mib.rtfd.io, and its web application is published at https://smile-mib.cs.queensu.ca.

In the following subsections, we describe the core learning algorithm in SMILE, discuss the metabolite importance and interaction assessment approach, and show the utilization of SMILE web application.

#### Evolutionary algorithm

Evolutionary algorithms define a collection of meta-heuristic optimization and modelling algorithms inspired by natural evolution [28]. An evolutionary algorithm maintains a population of diverse candidate solutions to a problem. An initial set of candidate solutions are often generated randomly. Each new generation is produced by probabilistically selecting better solutions for reproduction, and introducing small stochastic changes using biologically inspired operators such as mutation and crossover. Evolutionary computing has been successfully applied to machine learning problems, where it can automatically derive a symbolic predictive model. Such a variant of evolutionary algorithms was proposed as genetic programming [29], and has been used to solve classification and regression problems.

The evolutionary algorithm we used in this research is linear genetic programming (LGP) [30, 31]. LGP represents candidate predictive models in an evolutionary population using an imperative program. The fitness of a predictive model is defined as its classification accuracy. A population of diverse candidate models are initialized randomly and will improve fitness gradually through a large number of generations. After evolution, we obtain a best evolved model with the highest fitness score.

**Fig. 1:**
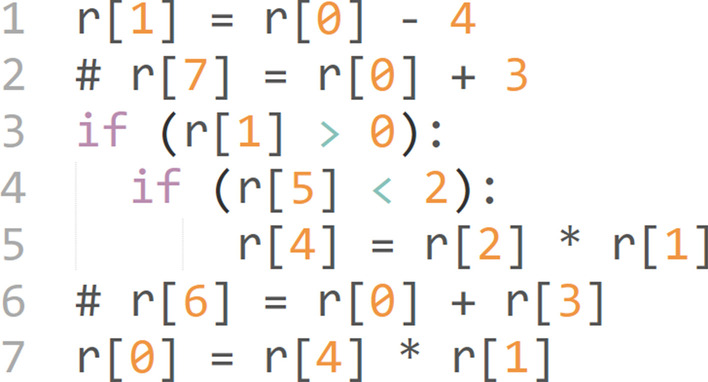
Representation of an LGP program. This example program has seven instructions, which will be executed in a sequential order. An instruction can be an assignment statement or an *if* statement. Registers are used to store input variables and to perform computation. *r*[1] to *r*[5] are calculation registers and *r*[6] to *r*[10] are input registers. Register *r*[0] is the designated output register and its final value after the execution of all instructions will be the output of this program

Similar to an imperative program, an LGP model consists of several instructions. Each instruction is either an assignment or a conditional statement. An assignment statement has three registers, i.e., one return register and two operand registers. For instance, in the LGP program shown in Fig. 1, instruction 1 assigns the value of *r*[8] minus a constant 4 to *r*[1]. The set of instructions are executed sequentially. The conditional *if* statement controls the program flow. If the condition is true, the subsequent instruction is executed, otherwise the subsequent instruction is skipped. In case of nested *if* statements, all conditions need to be true for the subsequent instruction to be executed. For example, in Fig. 1, line 5 is executed only if the conditions in line 3 and line 4 are both true. Register *r*[0] is the designated output register, and its final value after execution will be projected using a Sigmoid function to classify a sample either as diseased or healthy.

Note that not all instructions modify the final value stored in *r*[0]. We define an effective instruction as one that contributes to the final output, and a non-effective instruction otherwise, e.g., line 2 and line 6 in Fig. 1.

A register stores the value of a variable. There are two types of variables in our LGP programs, the input variables and the calculation variables. Input variables are predictive features, i.e., metabolite concentrations, in this work. A calculation variable is used as a buffer to enhance computation capacity. The designated output register *r*[0] is a special calculation register. Constants are chosen from a user-defined interval. Furthermore, a return register, i.e., the one on the left side of an assignment, can only be a calculation register. In this way, our method inherently prevents overriding of the input feature values.

In each generation, parent models are chosen using a tournament selection, i.e., a randomly chosen set of models compete and the fittest two will be picked to reproduce. To these selected parents are then applied genetic operations including crossover, macro-mutation, and micro-mutation with a certain probability. Crossover combines the genetic information of two parents to generate two new offspring. Two crossover points are picked randomly in each parent model, the instructions defined by the two points are swapped between two parent models. Macro-mutation insert or delete an instruction in a model. Micro-mutation randomly picks an instruction in a model and changes either a register or the operation in that instruction.

**Fig. 2:**
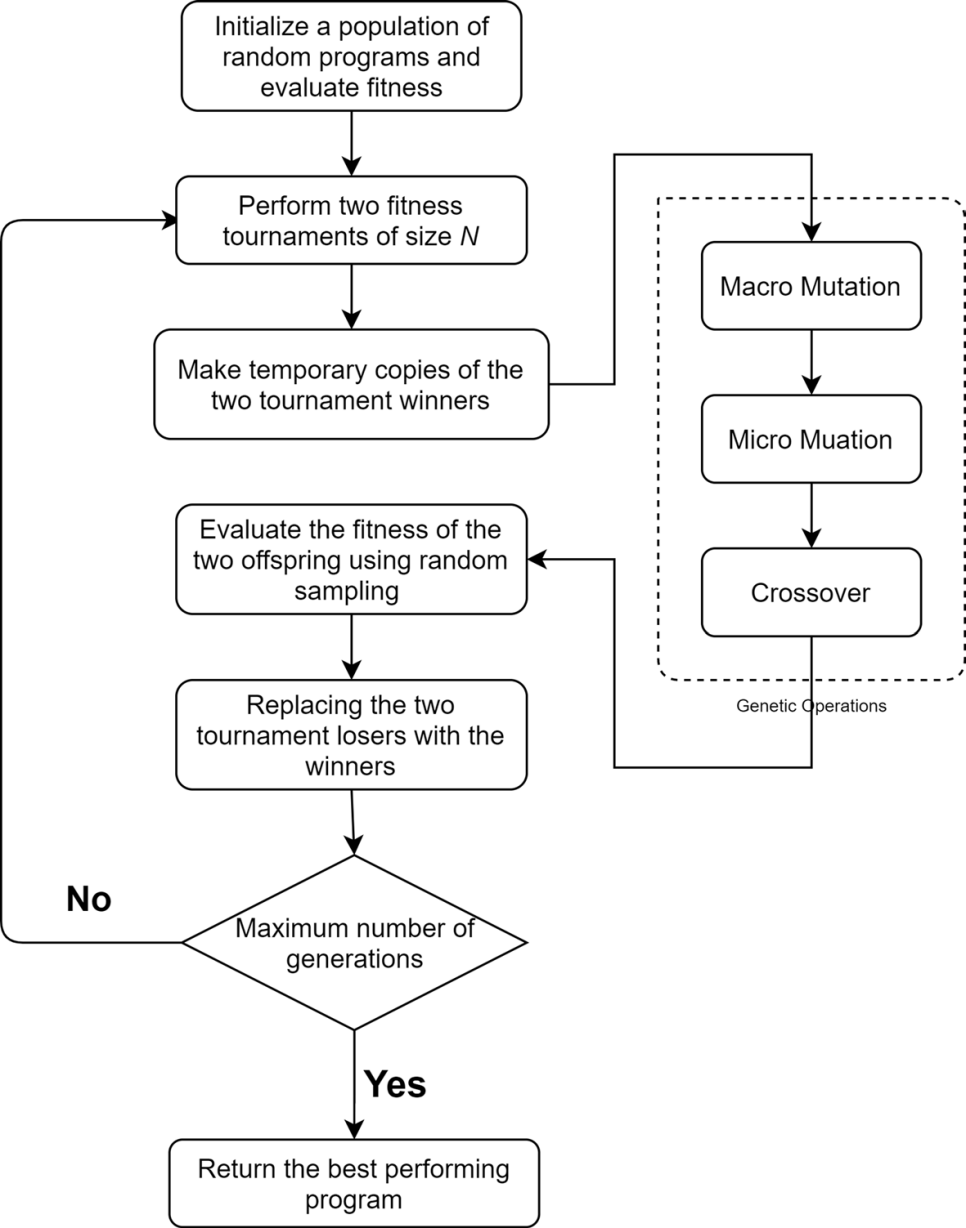
LGP algorithm flowchart. After initialization, this evolutionary algorithm repeats the processes of parent selection, mutation, crossover, and replacement, until the generation limit has been reached

Then, the two new offspring replace the worst two models in the tournament, and their fitness values are computed. In each run of this evolutionary algorithm, this process is repeated until the limit of the number of generations is reached. The model with the highest fitness score will be saved as a result of evolution. A flowchart of our LGP algorithm is shown in Fig. 2.

**Table 1:** Parameter configuration in the LGP algorithm

Parameter	Setting
Fitness function	Accuracy score
Program initialization	Random
Program initialization length	[10, 40]
Max program length	300
Population size	800
Number of generations	240
Operator set	+, -, x, $\div$, exponent, $if<$, $if>$
Constant set	Random int from 1 - 10
Parent selection	Tournament with size 8
Crossover probability	0.75
Micro-mutation probability	0.5
Macro-mutation probability	0.75
Random sampling	Bootstrap without replacement

Due to the stochastic nature of evolutionary algorithms, each run may yield a different resulting best model. We collected 1000 independent runs of this LGP algorithm. The main parameters used are shown in Table. 1. We randomly partitioned the data into a training set ($80\%$) and a testing set ($20\%$) and used a different random seed for each independent run of the algorithm. The fitness value of a model is computed as the training classification accuracy. In order to prevent overfitting and to reduce the computational overhead of fitness calculation, we used bootstrapping and sampled 50–100% of the training set, without replacement, each time when computing the fitness of an individual model. The final best evolved predictive model of each run is then evaluated using the testing set. The testing accuracy and other prediction performance metrics are thus computed using unseen testing samples unique to each evolved best model.

#### Feature importance and interaction analysis

Note that input features, i.e., metabolite concentrations, are stored in registers in an LGP program. In the initialization of the program population, some input registers may be chosen in an instruction of a program, and some may not. In addition, an input register may be mutated or lost as a result of the mutation and crossover operations. An input register may also become ineffective when its value does not contribute to the calculation of the final value of *r*[0]. For instance, in Fig. 1, removing input register *r*[7] does not alter the final value of *r*[0], thus it is considered as an ineffective register, and its represented feature is considered as an ineffective feature. Therefore, the selection of effective metabolite features is embedded in the LGP algorithm and co-evolved with predictive models.

We can rank individual features based on their occurrence frequencies in the collected 1000 best evolved models. This ranking provides a means to assess feature importance, i.e., if a metabolite feature most frequently appears in the best evolved models it may have a strong influence on explaining the prediction of the disease status.

In addition, if two features tend to co-occur frequently in a same best evolved model, they may have a strong synergistic interaction effect associated with the disease. We calculated this co-occurrence frequency for all pairs of features. Subsequently, we can construct a metabolite synergy network by including the top metabolite pairs that show the strongest synergistic interactions. These most frequently co-occurring metabolite pairs are represented as edges and their two end points. Such a network can help us visualize a large collection of pairwise feature interactions, and identify important metabolites that interact with many others.

#### Algorithm implementation

To facilitate a wider adoption of our proposed approach, we published all the source code of implementing our algorithm. For a robust prediction result and a comprehensive feature analysis, we recommend to collect a large number, e.g., 1000, of independent runs of the LGP algorithm. This can in turn require high computational power.

For the implementation and analysis included in this study, we used a large-scale high-performance computer cluster, Graham, from Compute Canada. We ran an array of 1000 jobs in parallel. Each job (an independent run of SMILE) took 8–10 hours and up to 500 MB memory running on one CPU core (Intel Xeon CPU E5-2667 v4 3.2 GHz).

After an individual job is completed, the result can be saved via calling the $save\_model()$ method. This method uses Python *pickle* module to implement object serialization. This will generate a .*pkl* result file that can be uploaded to the web interface later for interpretation and visualization. The web application also requires an original dataset .*csv* file. Users need to format the .*csv* file where rows are samples and columns are features (metabolite concentrations). The file header is the metabolite names and the first row is the class label (named “category”). Users can check formatting errors using an automated python file on SMILE’s Github page. Finally, users can upload the .*pkl* and .*csv* files to our web application.

#### Web application

We developed a web interface, https://smile-mib.cs.queensu.ca, for interpreting and visualizing the analysis results. First, a testing accuracy filter is provided for the user in order to include only the best-performing evolved models among all collected final evolved models by running the algorithm independently for 1000 times.

There are three modules for the result interpretation and visualization. The first module is *Feature Importance Analysis*. Users can decide to investigate LGP models with a specified number of effective features. Then, features are ranked based on their individual occurrence frequencies and showed in the “Feature Occurrence” graph. Clicking a feature of interest in this graph will show all LGP models containing that feature in the “Model Accuracy” graph. Further selection of a point in this graph will show its represented model in “Detailed Model Info” panel. This allows users to investigate and interpret a selected predictive model based on its testing accuracy and metabolite features involved.

Upon selecting the “Pairwise Co-occurrence Analysis” panel, users can see a heat map of “Feature Pairwise Co-occurrence”, which shows all the pairwise co-occurrence frequencies in the selected LGP models. Moreover, users can manually choose a pair of features to see their distributions in diseased cases and healthy controls in “Two-Feature Scatter Plot”.

The second module is *Co-occurrence Network Analysis*. Users can visualize a network of the top most common metabolite pairs. In this graph visualization, a node is a metabolite and an edge links two metabolites if their co-occurrence frequency is above the top threshold. The node size is proportional to individual feature’s occurrence frequency. The edge width is proportional to pairwise co-occurrence frequency, which is also labeled on each edge.

Users can also investigate a metabolite/feature of particular interest. The third module of SMILE is *Search a Feature*. This module allows users to enter the name of a specific feature, and will show this feature’s individual occurrence frequency and its interacting features, ranked by their co-occurrence frequencies. In addition, SMILE provides a visualization of the synergy sub-network of this feature that includes all its directly interacting neighbours.

## Results

### Best evolved classification models

**Fig. 3:**
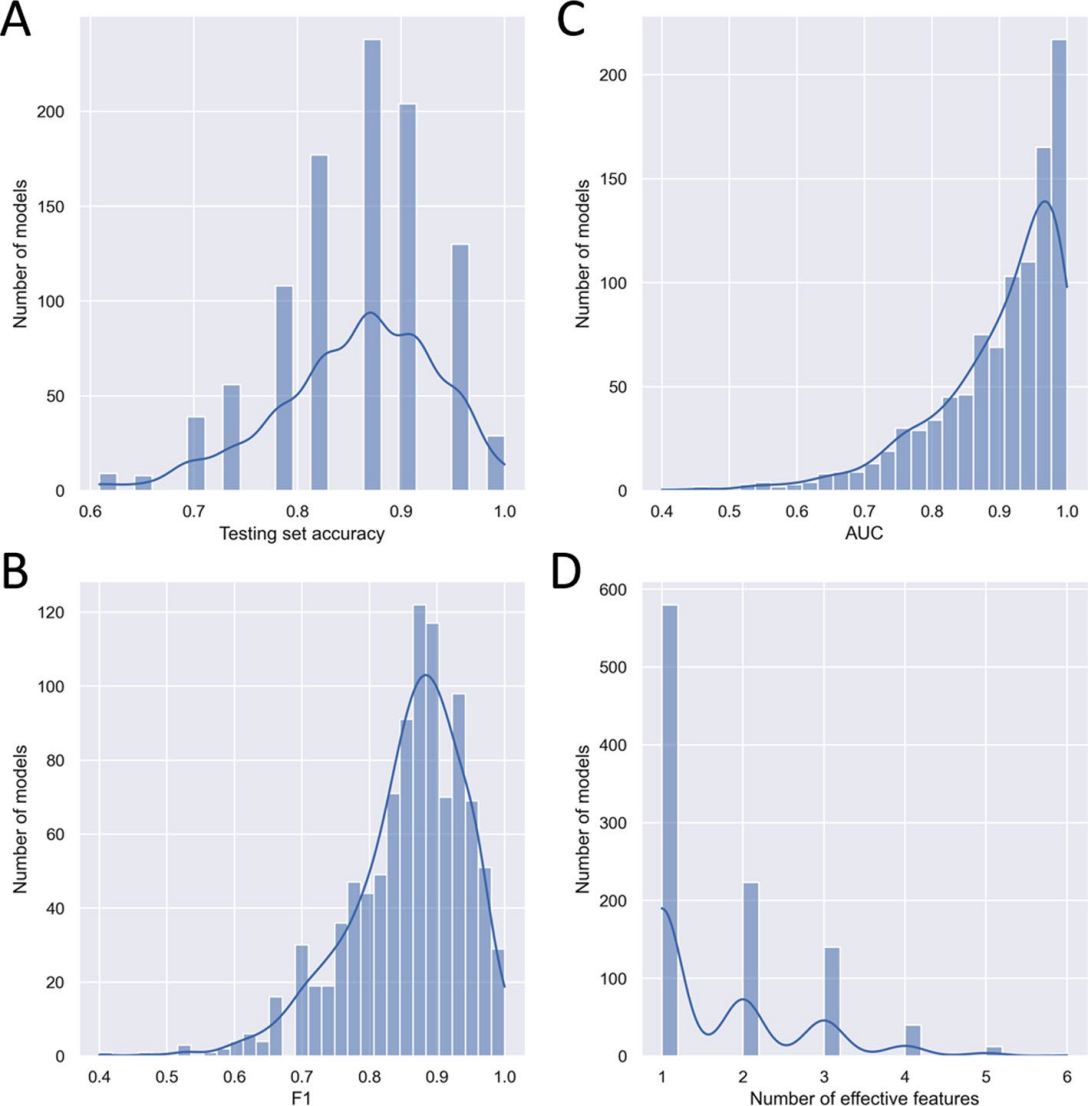
Classification performance of applying SMILE to the AD metabolomic data. The figure shows the distribution of the 1000 evolved best models in terms of **A** testing accuracy, **B** $F_1$ score, **C** AUC score, and **D** number of effective features

For the determination of significant metabolites and metabolite interactions in AD and aMCI we ran our algorithm 1000 times and collected 1000 evolved classification models for each of the three pairwise comparisons among AD patients, aMCI patients, and healthy controls. In this section, we discuss the result of comparing AD patients with healthy controls. Fig. 3 shows the classification performance of these 1000 models. Most of these models achieved a testing accuracy higher than 80%, i.e., they correctly classified 19 out of 23 testing samples.

The models were then evaluated using $F_1$ and AUC scores. The $F_1$ score can be interpreted as a weighted average of the precision ($\frac{\text {True Positive}}{\text {True Positive} + \text {False Positive}}$) and recall ($\frac{\text {True Positive}}{\text {True Positive} + \text {False Negative}}$). It is computed as $F_{1}=\frac{2 \times \text {precision} \times \text {recall}}{\text {precision} + \text {recall}}$. An $F_1$ score is between 1 (best) and 0 (worst). Receiver operating characteristic curve (ROC curve) shows the performance of a classification model at all classification thresholds. It uses false positive rate as the *x*-axis and true positive rate as the *y*-axis. Area under the ROC curve (AUC) measures the entire two-dimensional area under the ROC curve. A higher AUC value indicates a better classification performance of a model. The distributions of $F_1$ and AUC scores are shown in Fig. 3B, C. There are over 200 models, out of the 1000 we collected, that have a perfect AUC score of 1.0. Similarly, the majority of the models achieved an $F_1$ score greater than 0.8. These results suggest that the classification models evolved by our LGP algorithm are of very high quality.

We looked at the number of effective features in our evolved models. As shown in Fig. 3D, about 900 classification models use only 1–3 effective features. This suggested the existence of a few strong biomarkers that can effectively distinguish AD patients from healthy individuals in the data.

**Fig. 4:**
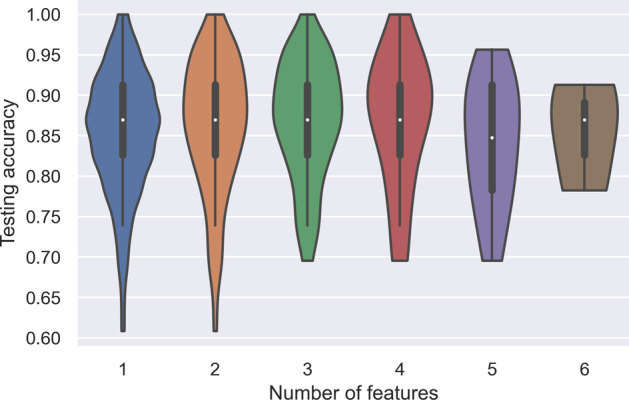
Model testing accuracy in relation to the number of effective features. For all models using a given number of effective features, a violin plot shows the distribution of their testing accuracy. The correlation between testing accuracy and the number of effective features is neither strong nor significant

Then, we investigated the correlation between the number of effective features and the testing classification accuracy. Fig. 4 shows the violin plot of model testing accuracy in relation to the number of effective features used in a model. The observed correlation is neither strong nor significant. (Pearson’s correlation $r=0.049$, $p=0.123$).

### Most important features and interactions

**Fig. 5:**
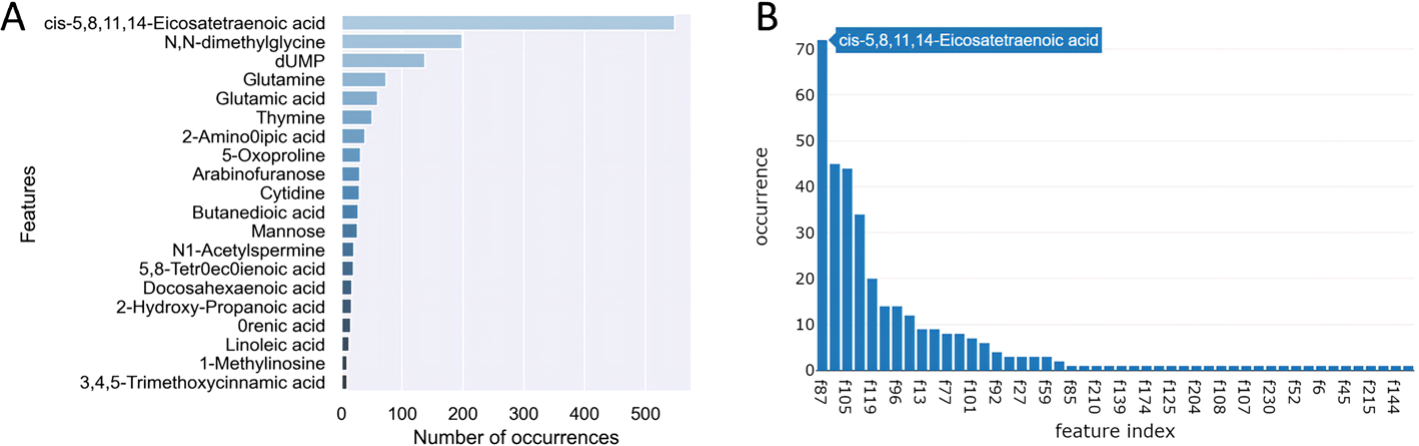
Individual feature importance ranking. **A** The overall feature importance ranking using 1000 evolved models. **B** A feature occurrence graph generated on SMILE’s web interface using 176 filtered models with a higher than 80% testing accuracy and using two effective features. The *x*-axis shows feature index and *y*-axis shows feature occurrence frequency. The corresponding feature name will be shown with mouse hover

Figure 5A shows the top 20 individual features based on their occurrence frequencies in the 1000 best evolved models. The top metabolite cis-5,8,11,14-Eicosatetraenoic acid appears in about 55% of all models. N,N-dimethylglycine and dUMP are also found in approximately 20% and 15% evolved models, respectively. In addition to these three top ranked individual metabolites, glutamine, glutamic acid, thymine, 2-Aminoadipic acid were found in more than 5% of the evolved models. SMILE also allows us to filter the collected 1000 evolved models using a threshold of testing accuracy and the number of effective features in a predictive model. For instance, Fig. 5B illustrates the feature occurrence ranking using 176 models that have a testing accuracy higher than 80% and include two effective features.

**Fig. 6:**
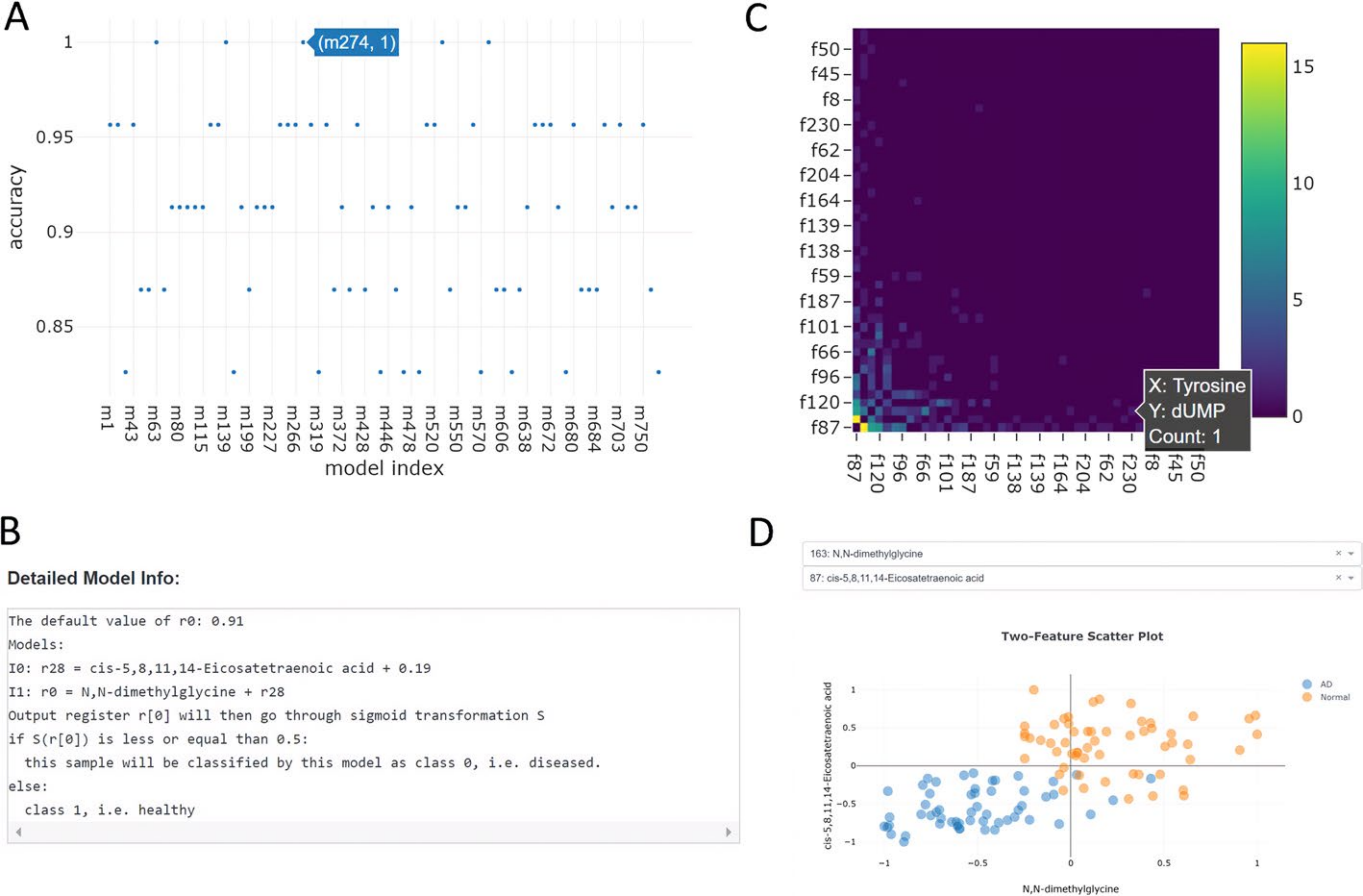
Interpretable learning results of SMILE on the AD metabolomic data. All sub-figures are generated by SMILE web interface. **A** Model accuracy graph shows the testing accuracy of all predictive models that use a chosen feature by clicking a bar in Fig. 5B. This graph, for instance, shows the testing accuracy of models that use cis-5,8,11,14-Eicosatetraenoic acid as an effective feature. **B** Detailed model figure provides symbolic representation of a selected model, by clicking a point in **A**. **C** Feature pairwise co-occurrence graph represents the frequencies using a heat map, and provides the metabolite names though mouse hover. **D** Two-feature scatter plot depicts the distribution of the two chosen metabolites’ concentrations. The metabolite pair shown in this figure has the highest co-occurrence frequency in the AD metabolomic data

Clicking on the most important feature (cis-5,8,11,14-Eicosatetraenoic acid) in Fig. 5B will show all models containing this feature in Fig. 6A. Further clicking on a point (e.g., model m274) in this graph will show the detailed predictive model in Fig. 6B. This model has a testing accuracy of 1, contains two instructions, and uses metabolites cis-5,8,11,14-Eicosatetraenoic acid and N,N-dimethylglycine as effective features. Essentially, a prediction is made by comparing the sum concentration of the two metabolites to a constant.

Figure 6C shows the heat map of pairwise co-occurrence frequencies (the “Feature Pairwise Co-occurrence graph” on the website). Upon clicking on a cell in this graph, the distributions of the two corresponding metabolites in the populations are shown in a scatter plot (Fig. 6D). Of the AD metabolomic data used in this study, metabolite pair N,N-dimethylglycine and cis-5,8,11,14-Eicosatetraenoic acid, combined linearly, are able to clearly distinguish AD cases and healthy controls. This was also evidenced in the discovered predictive model shown in Fig. 6B.

### Feature co-occurrence network

**Fig. 7:**
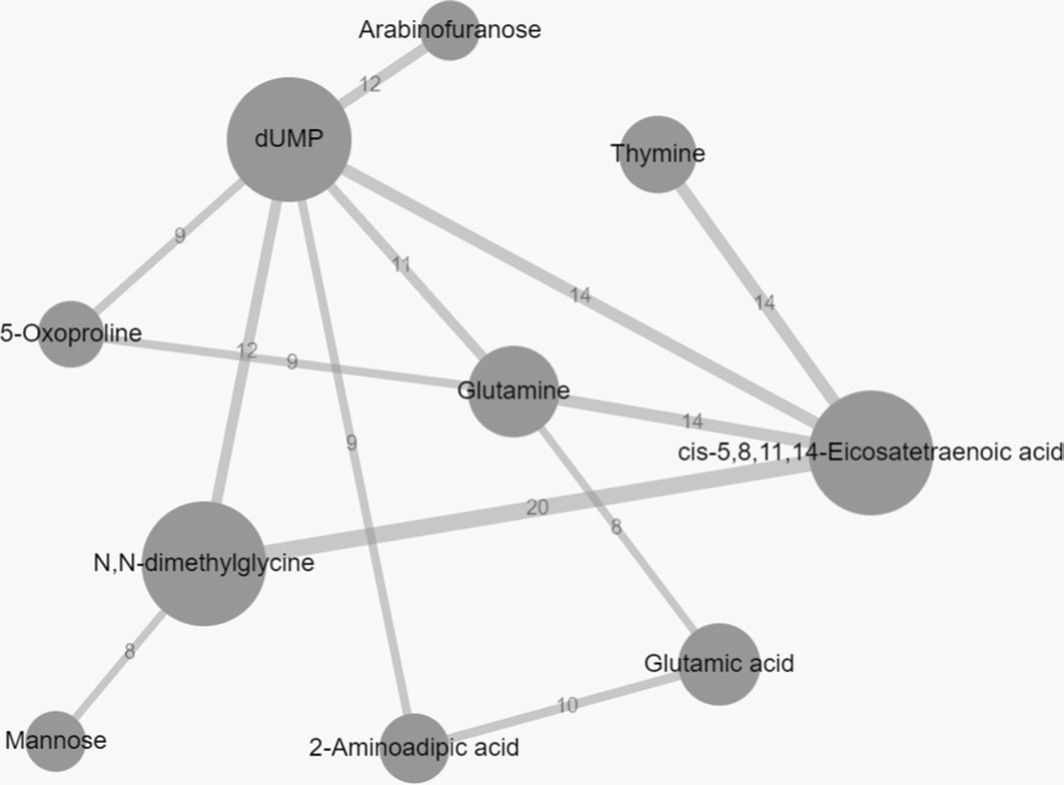
Synergy network of top 3% most common metabolite pairs in the AD metabolomic data. Metabolite pairs with the most frequent co-occurrences are represented as edges and their two end points. The edge width is proportional to pairwise co-occurrence frequency, also labeled on each edge. The vertex size is proportional to individual feature’s occurrence frequency

The top 3% most common metabolite pairs were used to construct a synergy network (shown in Fig. 7). Here, a vertex is a metabolite and its size is proportional to the corresponding metabolite’s individual occurrence frequency in the selected evolved models. The most important metabolites (see Fig. 5A), such as cis-5,8,11,14-Eicosatetraenoic acid, dUMP, and N,N-dimethylglycine, also appear in this network as larger vertices. An edge links two metabolites if their co-occurrence frequency is among the top 3%. The edge width is proportional to the co-occurrence frequency of a metabolite pair, which is also shown as the edge weight (label).

Specifically, metabolite dUMP has 6 edges with an average edge weight of 11. In addition, cis-5,8,11,14-Eicosatetraenoic acid has strong connections with four other metabolites. The strongest synergistic interaction between cis-5,8,11,14-Eicosatetraenoic acid and N,N-dimethylglycine, found in the AD metabolomic data, is also shown in this network.

### Comparative studies

To further evaluate the classification performance and feature importance assessment of our algorithm, we compared its results with two widely-used learning and feature analysis algorithm, random forest (RF) and support vector machine (SVM).

We implemented the RF algorithm with five-fold cross-validation using the *random forest classifier* and *cross validate* packages in *Scikit-learn* [27]. The RF hyper-parameters were optimized using grid search. It exhaustively searches a manually specified subset of the hyper-parameter space. The classifier with its hyper-parameter configuration that performs the best on the validation data will be chosen by the search. We used grid search to optimize RF hyper-parameters “max_depth”, “min_samples_split”, “n_estimators”, and “max_features”. The optimized RF parameter values as well as other configurations were shown in Additional file 1: Table 2. Similarly, we performed grid search to optimize SVM hyper-parameters “C” and “gamma”, and the final parameter values were shown in Additional file 1: Table 3.

**Fig. 8:**
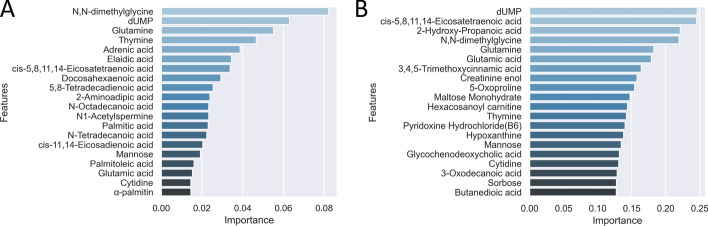
Feature importance ranking using RF and SVM. **A** Metabolite features are ranked based on the Gini importance in RF. **B** Metabolite features are ranked based on the linear kernel weight in SVM

The feature importance ranking is the average feature importance over five validation groups (shown in Fig. 8A). The $F_1$ scores in each validation groups are $\{1, 0.95, 1, 0.95, 1\}$. The metabolites are ranked based on their Gini importance scores and the top 20 metabolites are shown in the figure. Metabolites dUMP and N,N-dimethylglycine are ranked among the top three by both RF and SMILE. There are 10 common metabolites among the top 20 ranked by both algorithms. Discrepancy can also be observed. For instance, metabolite 5-Oxoproline is ranked 8th by SMILE but absent in the top 20 by RF. This metabolite is also captured in the synergy network through strong interactions with dUMP and Glutamine (see Fig. 7).

We also implemented the linear kernel SVM with five-fold cross-validation using the *support vector classification* and *cross validate* packages in *Scikit-learn* [27]. The feature importance ranking is based on the average feature importance over five validation groups (shown in Fig. 8B). The $F_1$ scores in each validation groups are $\{1, 1, 0.96, 0.96, 0.91\}$. The importance of a metabolite is inferred based on its coefficient or weight in the linear kernel. Metabolites dUMP and cis-5,8,11,14-Eicosatetraenoic acid are ranked as the top two by SVM and among the top three by SMILE. On the other hand, the 8th ranking metabolite creatinine enol by SVM does not appear in the top 20 list by SIMILE or RF.

## Discussion

Our bioinformatics methodology and software SMILE demonstrates a great potential of applying interpretable machine learning to biomedical science. SMILE uses an evolutionary algorithm for the discovery of key metabolites and provides an interactive website for result visualization and interpretation. The evolutionary algorithm is able to train a large collection of high-performing predictive models, represented as computer programs. These programs are a compact set of instructions and can be easily interpreted, so the mechanistic explanation on a prediction can be transparent to an end-user. SMILE also provides a means to evaluate feature importance and feature interactions since selecting the most relevant features (metabolites) are embedded in the algorithm and co-evolved with predictive models. SMILE uses networks to visualize the importance of features and their interactions.

We demonstrate the powerful utility of SMILE by applying it to an AD metabolomic dataset [26]. SMILE was able to find compact predictive models using few metabolites with a high accuracy. This may be explained by the fact that the data were collected from late-stage AD patients, and using only a small number of key metabolites was able to clearly separate AD patients from healthy controls. Nevertheless, SMILE focused on producing interpretable learning results and indeed provided new insights into influential metabolites and their interactions.

SMILE identified many key metabolites that have been previously linked to AD or are less researched but can be potentially linked to AD. Cis-5,8,11,14-Eicosatetraenoic acid (i.e. arachidonic acid), the most important metabolite found by our algorithm, is increased during neuroinflammation in the brain [32]. Arachidonic acid is highly prevelant polyunsaturated fatty acid in the brain with high presence in membranes primarily in its esterified form. Free arachidonic acid plays a major role in neuroinflammatory response through conversion into pro-inflammatory eicosanoids [33] with role both in clearing the amyloid-beta plaque and increasing production of neurotoxic compounds. Additionally, free arachidonic acid acts as a retrograde synaptic messenger and a regulator of neuromediator exocytosis. Finally, it is an activator of kinases involved in tau hyperphosphorylation. Arachidonic acid usually has an increased concentration in AD patients’ brain especially in the high densities of senile region with activated microglia. The senile plaques are infiltrated by activated microglia secrete inflammatory cytokines, where an increased expression of enzymes cPLA2 and sPLA2 leads to more inflammatory arachidonic acid. Increased blood concentration of Arachidonic acids, together with changes in concentrations of other unsaturated and polyunsaturated fatty acids have been linked to neurological diseases including dementias [34]. Number of other fatty acids have been selected by our method as highly diagnostic for AD as well as aMCI including for example docohexaenoic acid (DHA) with known role in AD prevention and development [35]. N, N-dimethylglycine, second most significant metabolite in our analysis, is part of glycine, serine threonine metabolic pathway highly relevant for metabolism of choline, sarcosine, methhionine and betaine all of major importance in AD [36]. At the same time N,N-dimethylglycine in combination with glutamate, glycine and its N-methyl derivatives was shown to increase frequency and amplitudes of the NMDA receptor-mediated excitatory field potentials [37]. Importantly both glutamate (glutamic acid) and glutamine are among the top metabolites ranked by SMILE. It is well known that the glutamate-glutamine cycle between neurons and astrocytes requires an adequate supply of the neurotransmitter glutamate. Study found a drop in the glutamate/glutamine ratio in AD and aMCI patients [38].

In many bioinformatics data, features can correlate given the intertwined relationships of entities in complex biological systems [39]. In the AD metabolomics data, it is also plausible that metabolites are involved in the same biochemical reactions and their concentrations in a sample correlate. We performed pairwise feature correlation analysis (see Additional file 1: Fig.8) and found 61 pairs of metabolites that have a Pearson’s correlation coefficient greater than 0.8 and a *p*-value less than 0.05. These metabolite pairs are listed in Additional file 1: Table 1. None of these highly correlated pairs showed up together in the top 20 list by SMILE. However, the correlated pair N,N-dimethylglycine and 2-Aminoadipic acid ($r=0.91$, $p<10^{-50}$) were ranked 1st and 6th by RF.

In addition to comparing AD patients with healthy population, we performed the same analysis comparing aMCI with AD, as well as comparing aMCI with healthy controls. The evaluation results of aMCI versus controls are shown in Additional file 1: Fig.1, Fig.2, and Fig.3. The majority of the evolved models achieve an AUC score greater than 0.8 and a $F_1$ score greater than 0.75. In the feature importance graph Additional file 1: Fig.3, the top 10 metabolites are very similar to that of comparing AD patients with healthy controls. The top metabolite cis-5,8,11,14-Eicosatetraenoic acid, identified previously, also shows in $\sim$50% of the evolved models. Metabolites dUMP and thymine have increased importance, ranked second and third when comparing aMCI with controls. We also compared the performance of our approach with that of RF and SVM studying aMCI versus healthy controls. The feature importance rankings by RF and SVM are shown in Additional file 1: Fig.4.

Using the SMILE result of comparing aMCI with AD, the top 3% most common metabolite pairs were shown in a synergy network (shown in Additional file 1: Fig.5). Metabolites cis-5,8,11,14-Eicosatetraenoic and dUMP, the top two features interacting with the most other metabolites while comparing AD with controls, were also identified comparing aMCI with controls. Once again several other fatty acids have been determined as significant such as DHA linoleic acid as well as number of other poly-unsaturated and unsaturated fatty acids that have been indicated before for their role in AD development [40]. Metabolites dUMP and 2-Amionadipic acid have the strongest synergistic interaction with a co-occurrence frequency of 31.

Neither SMILE nor RF performed well when comparing AD with aMCI populations. Additional file 1: Fig.6 shows the classification result using SMILE. The AUC and $F_1$ scores are around 0.5. Additional file 1: Fig.7 shows the resting result of RF, which had an AUC score of 0.45. This indicates that the metabolites investigated in the dataset were not able to distinguish between AD and aMCI. This would suggest that in this cohort set aMCI population is further on the path of full AD development and possibly also explains observed similarity in markers between healthy versus AD and healthy versus aMCI.

## Conclusion

SMILE is an interpretable machine learning approach and can be a useful addition to metabolomics analysis tools. It is able to (1) evaluate both individual metabolite importance and pairwise interactions, and (2) evolve interpretable predictive models that provide insights into the underlying biochemical mechanisms. Most commonly used feature importance algorithms focus on ranking features separately but less on synergistic feature interactions. More powerful learning algorithms, such as deep neural nets, are able to produce highly accurate predictions but struggle to translate the learned knowledge embedded in the “black-box” models.

The limitation of our approach is the computational cost of running an evolutionary algorithm and collecting a large number of independent runs, since the algorithm maintains a large population of candidate predictive models. This can be alleviated by utilizing parallel computation and high-performance computing infrastructure.

Our methodology and software provide a novel bioinformatics framework for metabolomics. We make all our source code publicly available in order to benefit a wider research community and contribute to Python machine learning tool ecosystem. Our next steps include (1) generalizing this approach to data types other than metabolomics, and (2) improving our methodology in order to address common challenges in biomedical data analyses including high dimensionality, insufficient data samples, and hidden sub-types in complex diseases and disorders.

## Supplementary information

**Additional file 1**. Supplementary figures and tables.

## Data Availability

All source code of our approach is publicly available at https://github.com/MIB-Lab/SMILE, the detailed documentation of function usage in SMILE is avaliable at https://smile-mib.rtfd.io, and the web application can be accessed at https://smile-mib.cs.queensu.ca.
