# Supplementary material for: SMILE: systems metabolomics using interpretable learning and evolution
Source: BMC Bioinformatics. 2021 May 28;22:284. doi: 10.1186/s12859-021-04209-1 (PMC8161935; doi:10.1186/s12859-021-04209-1)
Supplement: Supplementary file 1 — Additional file 1. Supplementary figures and tables. [file 12859_2021_4209_MOESM1_ESM.pdf]

SMILE: Systems Metabolomics using Interpretable Learning and Evolution

Chengyuan Sha, Miroslava Cuperlovic-Culf, and Ting Hu

Supplementary files

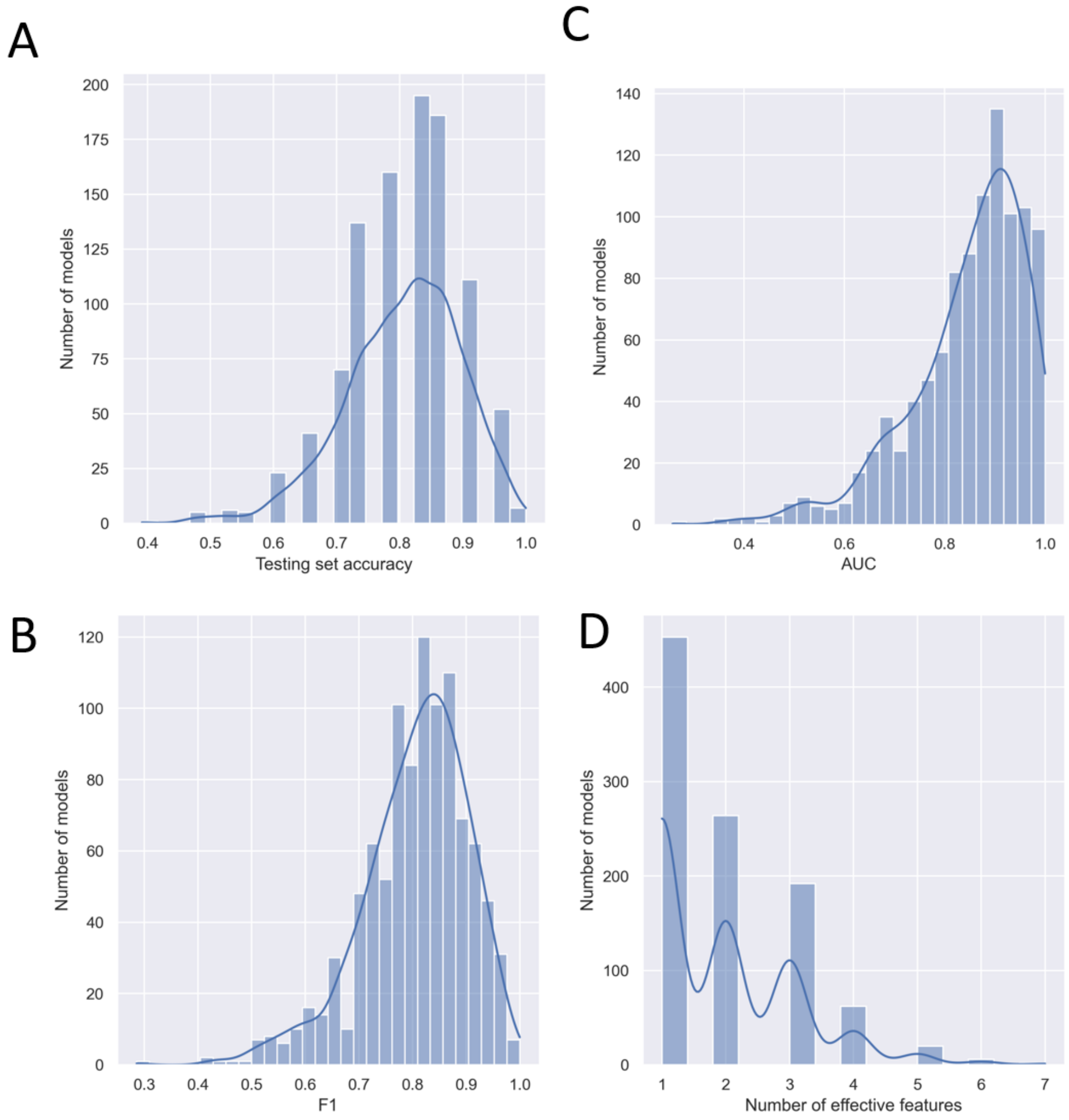

S_Fig. 1: SMILE classification performance comparing aMCI patients with healthy controls. The figure shows the distribution of the 1000 evolved models in terms of (A) testing accuracy, (B) F1 score, (C) AUC score, and (D) number of effective features.

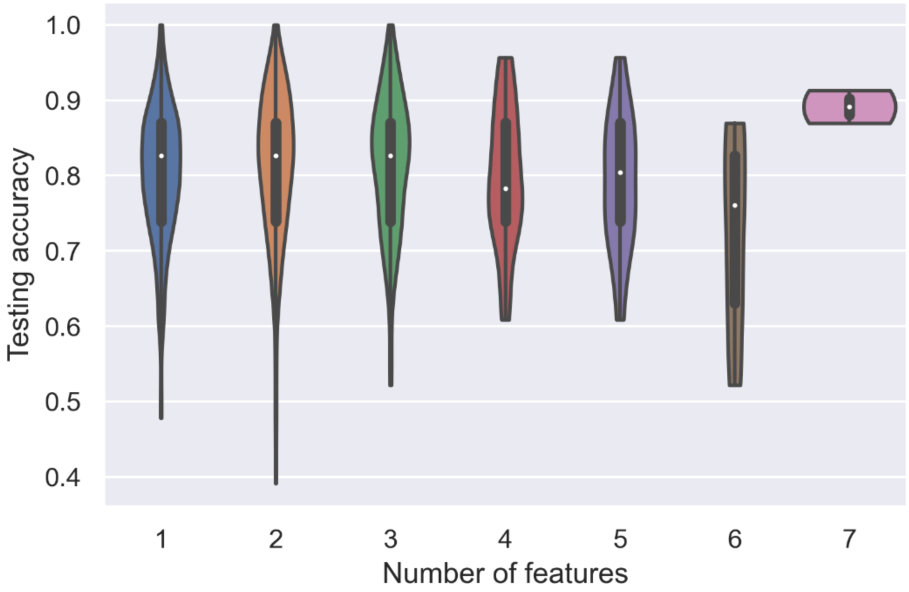

S_Fig. 2: Model testing accuracy in relation to the number of effective features (aMCI versus controls). No significant correlation was found.

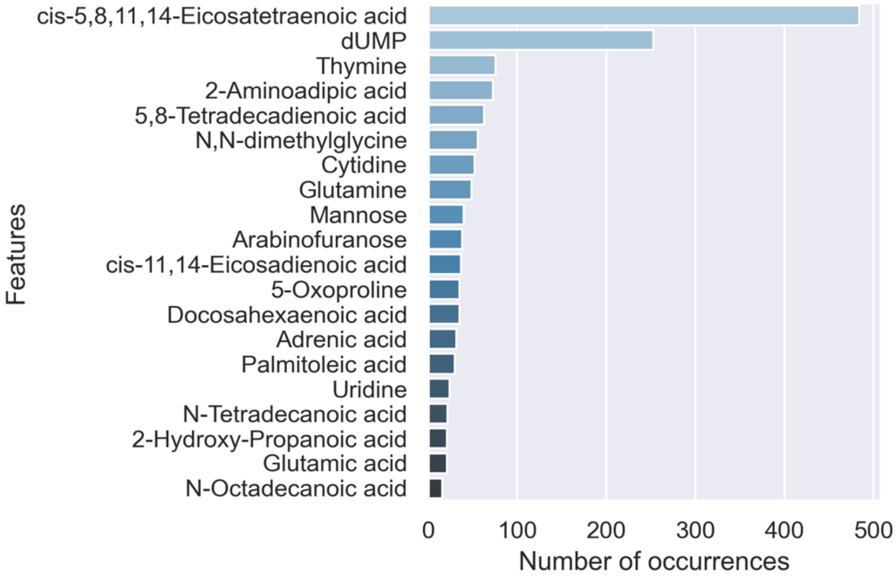

S_Fig. 3: Individual feature importance ranking (aMCI versus controls). The overall feature importance ranking was computed based on individual feature occurrence frequencies in the 1000 evolved best models.

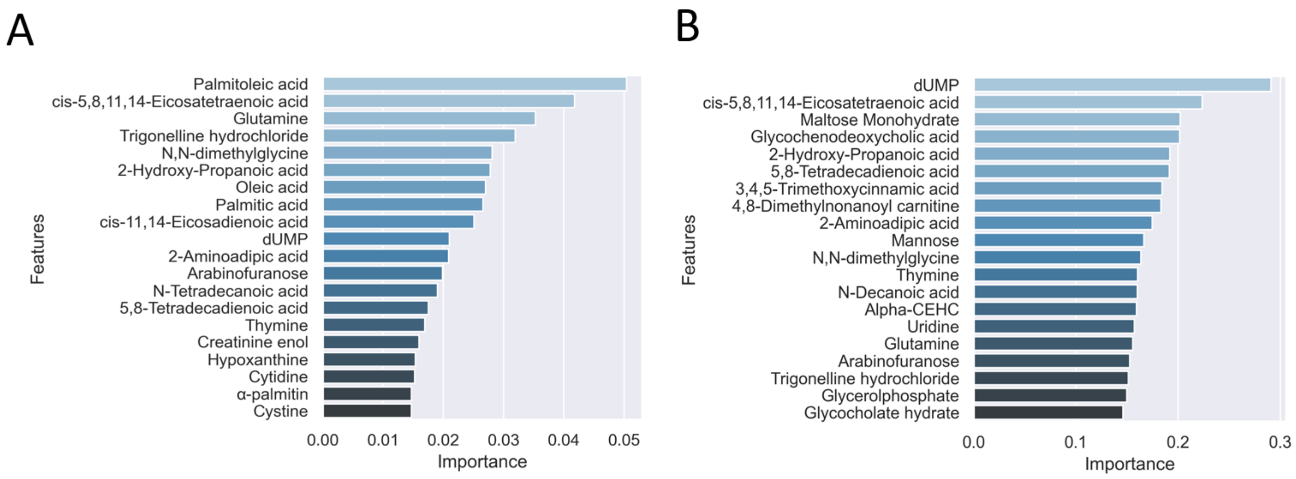

S_Fig. 4: Feature importance ranking using RF and SVM (aMCI versus controls). (A) Metabolite features are ranked based on RF Gini importance scores. (B) Metabolite features are ranked based on linear SVM coefficients.

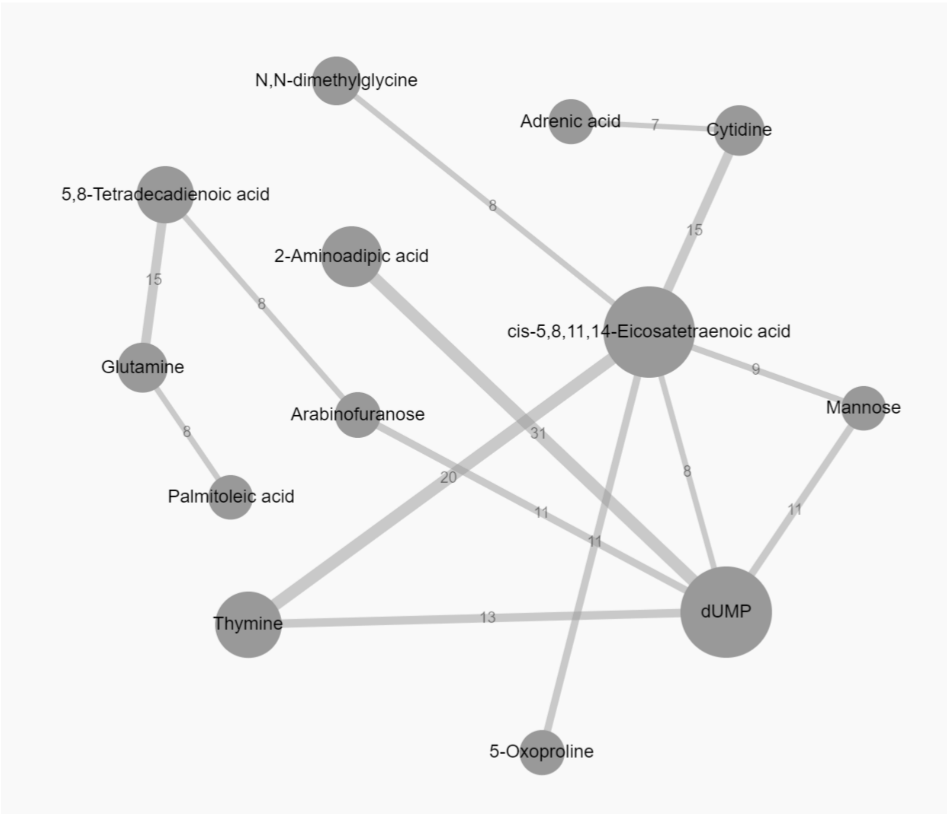

S_Fig. 5: Synergy network of top 3% most common metabolite pairs in the AD metabolomic data (aMCI versus controls).

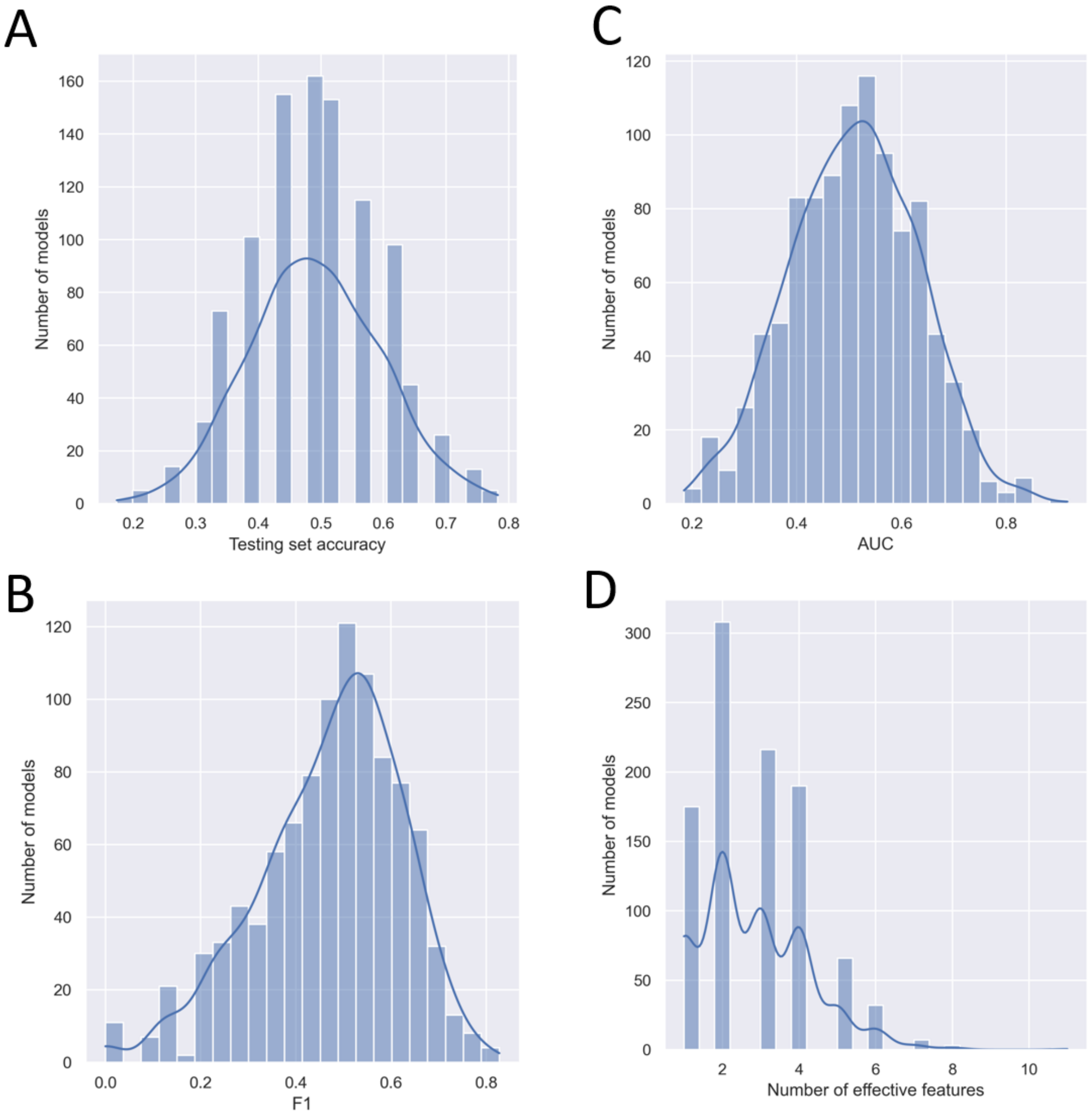

S_Fig. 6: SMILE classification performance comparing AD with aMCI patients. The figure shows the distribution of the 1000 evolved models in terms of (A) testing accuracy, (B) F1 score, (C) AUC score, and (D) number of effective features.

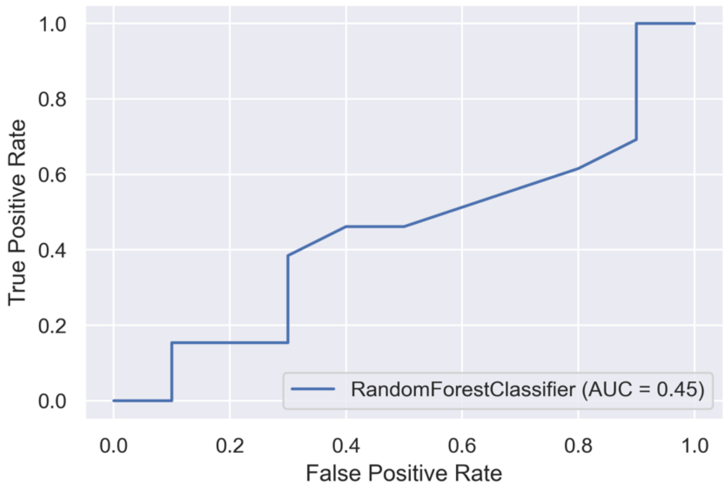

S_Fig. 7: ROC curve of RF prediction (AD versus aMCI).

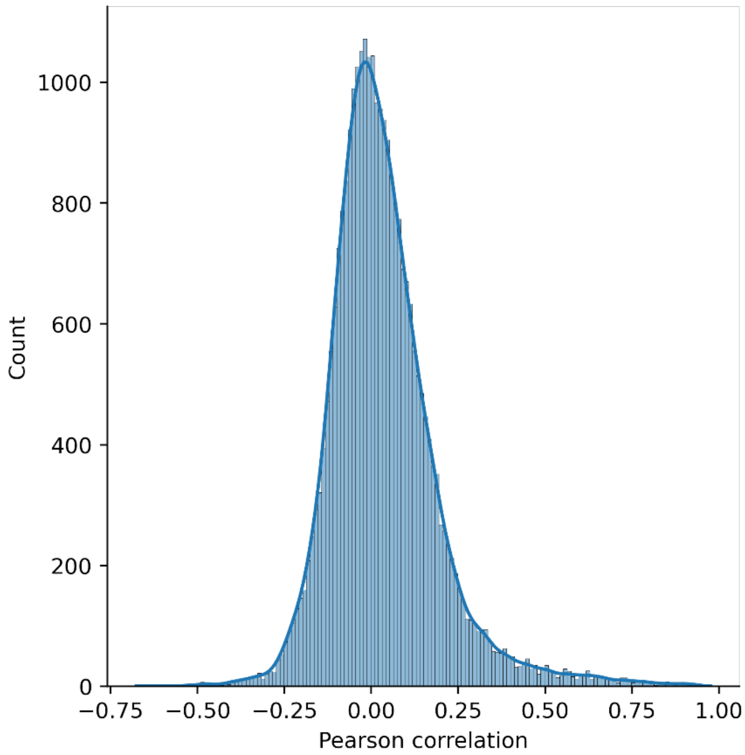

S_Fig. 8: The distribution of Pearson correlations of metabolite pairs (AD and controls).

S_Table. 1: List of highly correlated metabolite pairs (Pearson correlation $r > 0.8, p < 0.05$).

Pearson correlation	Feature 1	Feature 2
0.976502269	Stearoylcarnitine	Gluconate
0.951242527	4,8-Dimethylnonanoyl carnitine	2-Piperidinecarboxylic acid
0.942896593	N,N-dimethylglycine	Androsterone sulfate
0.939646387	Octanoylcarnitine	cis-5,8,11,14,17-Eicosapentaenoic acid
0.928890624	Lactose	Dopamine hydrochloride
0.925573236	Etiocholan-3A-ol-17-one crystalline	6-hydroxy-9H-Purine
0.924119752	N,N-dimethylglycine	4,8-Dimethylnonanoyl carnitine
0.923149716	Androsterone sulfate	2-Aminoadipic acid
0.922819641	Ubiquinone	Indoxyl
0.921689646	Androsterone sulfate	2-Piperidinecarboxylic acid
0.921320416	Octanoylcarnitine	Heptadecanoic acid
0.916834045	N,N-dimethylglycine	2-Piperidinecarboxylic acid
0.911292019	Octanoylcarnitine	Dopamine hydrochloride
0.910996497	N,N-dimethylglycine	2-Aminoadipic acid
0.910399542	2-Piperidinecarboxylic acid	2-Aminoadipic acid
0.902249233	Myo-Inositol	Lactose
0.90152178	Lactose	cis-5,8,11,14,17-Eicosapentaenoic acid
0.900529178	Dopamine hydrochloride	1H-Indole-3-acetamide
0.895315954	Androsterone sulfate	4,8-Dimethylnonanoyl carnitine
0.89338195	Lactobionic acid	Indoxyl
0.892642129	4,8-Dimethylnonanoyl carnitine	2-Aminoadipic acid
0.891304687	Norleucine	Heptadecanoic acid
0.890813037	Ubiquinone	Lactobionic acid
0.89030218	Heptadecanoic acid	cis-5,8,11,14,17-Eicosapentaenoic acid
0.890163694	Octanoylcarnitine	Lactose
0.884680815	Norleucine	cis-5,8,11,14,17-Eicosapentaenoic acid
0.878030502	Malonylcarnitine	2-Aminoacrylic acid
0.875246841	Lactose	1H-Indole-3-acetamide
0.873005389	Myo-Inositol	1H-Indole-3-acetamide
0.871135041	Tert-Butylaminotrimethylsilane	Taurine
0.86822598	Myo-Inositol	Dopamine hydrochloride
0.867685053	N-Dodecanoic acid	6-hydroxy-9H-Purine
0.856834086	Traumatic acid	Palmitoleic acid
0.855824319	Octanoylcarnitine	Norleucine
0.855239236	Octanoylcarnitine	1H-Indole-3-acetamide
0.854949206	Threitol	Cystathionine
0.854842933	Lactose	Cholic acid
0.852260905	N-Octanoic acid	Dopamine hydrochloride
0.850138633	Octanoylcarnitine	Myo-Inositol
0.84864652	Cholic acid	1H-Indole-3-acetamide

0.844009885	Dopamine hydrochloride	Cholic acid
0.843291439	Glycochenodeoxycholic acid	Glycine
0.839738466	Myo-Inositol	cis-5,8,11,14,17-Eicosapentaenoic acid
0.836757525	Palmitoleic acid	Indoxyl
0.83021994	Ubiquinone	Palmitoleic acid
0.829923495	Heptadecanoic acid	Dopamine hydrochloride
0.82898107	Hydroquinone	Alanine
0.827403085	Gluconic acid	5-Hydroxylysine hydrocholoride
0.82631061	Oxalic acid	Dopamine hydrochloride
0.825790096	Ubiquinone	Traumatic acid
0.824537656	Traumatic acid	Indoxyl
0.823705447	Dopamine hydrochloride	cis-5,8,11,14,17-Eicosapentaenoic acid
0.817672837	Myo-Inositol	Heptadecanoic acid
0.812775209	Indoleacrylic acid	Hydroxyacetic acid
0.812577352	Androsterone sulfate	1,3,7-Trimethyluric acid
0.812214596	Lactose	Heptadecanoic acid
0.809919733	Myo-Inositol	Cholic acid
0.805791046	Octanoylcarnitine	Cholic acid
0.80576235	N-Octanoic acid	Lactose
0.80188698	Palmitoleic acid	Lactobionic acid
0.800449106	cis-5,8,11,14,17-Eicosapentaenoic acid	Cholic acid

S_Table. 2: Parameters used in random forest classifier. Detailed parameter information can be found in sklearn.ensemble.RandomForestClassifier.

bootstrap	True
ccp_alpha	0.0
class_weight	None
criterion	Gini
max_depth	4
max_features	Sqrt (In AD versus controls)<br>Log2 (In aMCI versus controls)
max_leaf_nodes	None
max_samples	None
min_impurity_decrease	0.0
min_impurity_split	None
min_samples_leaf	1
min_samples_split	2
min_weight_fraction_leaf	0.0
n_estimators	50
n_jobs	None
oob_score	False
random_state	0
verbose	0
warm_start	False

S_Table. 3: Parameters used in Support Vector Classification. Detailed parameter information can be found in sklearn.svm.SVC.

C	0.1
break_ties	False
cache_size	200
class_weight	None
coef0	0.0
decision_function_shape	ovr
degree	3
gamma	1
kernel	linear
max_iter	None
probability	False
random_state	0
shrinking	True
tol	0.001
verbose	False
